# Use of the Minimally Invasive Reduction Instrumentation System for Facilitating Alignment and Reduction When Performing Minimally Invasive Plate Osteosynthesis in Three Dogs

**DOI:** 10.1155/2018/2976795

**Published:** 2018-04-15

**Authors:** Sarah Townsend, Daniel D. Lewis

**Affiliations:** Department of Small Animal Clinical Sciences, College of Veterinary Medicine, University of Florida, Gainesville, FL, USA

## Abstract

The Minimally Invasive Reduction Instrumentation System (MIRIS) was utilized to facilitate minimally invasive plate osteosynthesis (MIPO) of distal limb diaphyseal comminuted fractures (2 crural, 1 antebrachial) in three dogs. The MIRIS facilitated efficient MIPO in all three fractures. Radial and tibial lengths were restored within 2% of the length of the intact bone and postoperative frontal and sagittal plane angulation were within 3° of the normal contralateral limb for each of the fractures. Fixation failed in one of the tibial fractures when the plates bent a week following surgery. The implants were removed and the fracture was restabilized via MIPO facilitated by the MIRIS. Inappropriate implant selection was considered the primary reason for implant failure. All three fractures achieved union by 10 weeks following surgery. The dog that underwent revision surgery developed a surgical site infection 5 months following revision surgery, which necessitated implant removal. All three dogs had excellent limb function at the time of the final evaluation. This system resulted in reductions that were near anatomic, with acceptable restoration of length and alignment and excellent limb function.

## 1. Introduction

Minimally invasive plate osteosynthesis (MIPO) is utilized in both human and veterinary orthopedics and embraces the principles of biological fracture stabilization [1–9]. Iatrogenic soft tissue trauma and disturbance of the fracture environment are limited as implants are applied via small plate insertion incisions made remote to the fracture site [1–7]. Purported advantages afforded by this technique include reduced operative times compared to open anatomic fracture reconstruction [2, 10], low infection rates due to the shorter duration of surgery and limited exposure of the fracture site [8, 11–14], and shorter times to union ascribed to maintenance of the fracture hematoma and preservation of periosteal blood supply [15–17].

**Figure 1:**
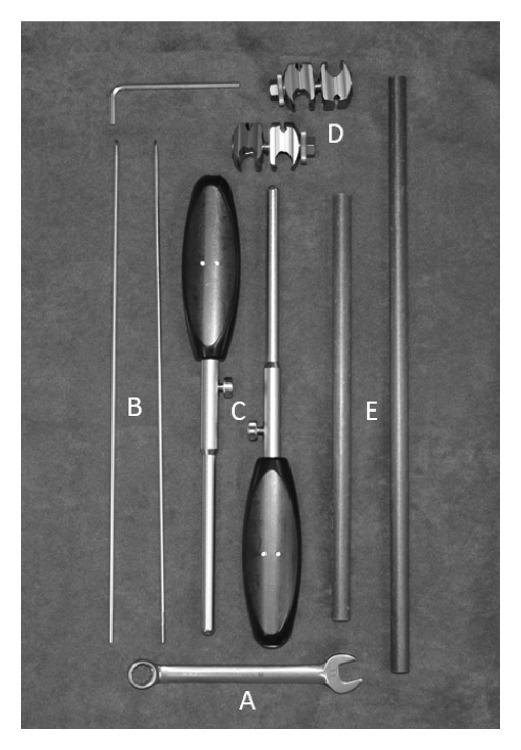
The Minimally Invasive Reduction Instrumentation System. A: 8 mm wrench; B: 2.8 mm partially threaded half-pins; C: cannulated reduction handles; D: connecting clamps; E: connecting rods.

Several indirect reduction techniques have been described to aid MIPO applications in dogs [7, 18–22]. The Minimally Invasive Reduction Instrumentation System [MIRIS] (DePuy Synthes, Paoli, PA) is a unilateral, linear fixator system marketed for use during MIPO applications in human patients (Figure 1). A recent canine cadaveric study was performed comparing the use of the MIRIS and a two-ring circular construct to facilitate alignment and reduction during MIPO applications using a comminuted radius and ulna fracture model [23]. The MIRIS allowed for shorter reduction times and simplified plate placement, without compromise to fracture reduction and alignment [23]. The objective of this case series was to report our initial clinical results using the MIRIS to facilitate MIPO applications in three dogs with diaphyseal appendicular fractures.

## 2. Case Description

**Figure 2:**
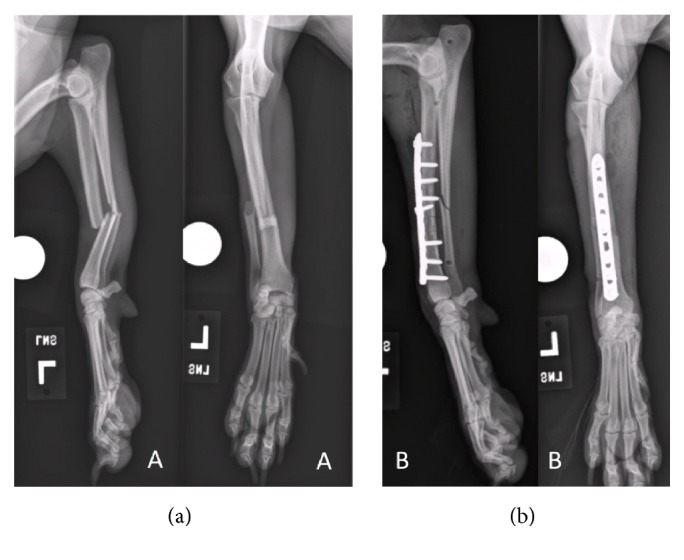
Craniocaudal and mediolateral preoperative radiographs of dog #1's left radius and ulna fractures (a). Initial postoperative radiographs following primary surgical stabilization with a 9-hole, 2.7 mm locking compression plate (b).

**Figure 3:**
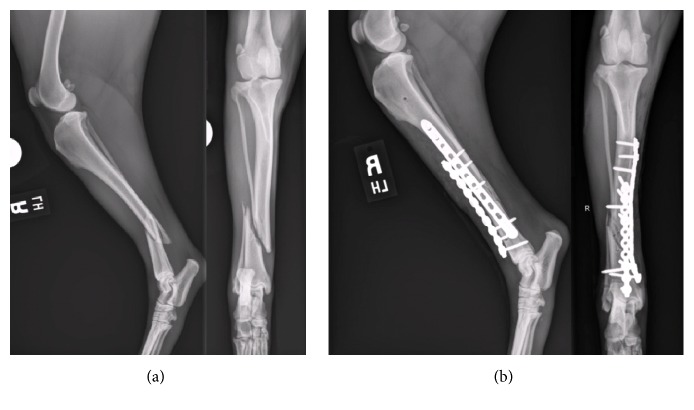
Craniocaudal and mediolateral preoperative radiographs of dog #2's right tibial and fibula fractures (a). Initial postoperative radiographs following primary surgical stabilization with a 12-hole, 2.7 mm locking compression plate and a 10-hole, 2.7 mm String of Pearls plate (b).

**Figure 4:**
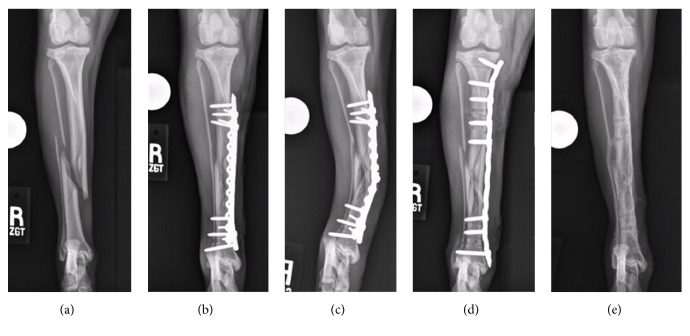
Craniocaudal preoperative radiographs of dog #3's right tibial fracture (a). Initial postoperative radiographs following primary surgical stabilization with a 12-hole, 2.7 mm locking compression plate and a 10-hole, 2.0 mm String of Pearls plate (b). Implant failure was documented 7 days after surgery (c). The fracture was restabilized via application of an 11-hole, 3.5 mm LCP plate (d). Radiographs obtained 3 weeks after implant removal necessitated by infection (e).

Three dogs were presented for stabilization of long bone fractures (Table 1). All dogs had closed diaphyseal spiral fractures: one radius and ulnar fracture (Figure 2) and two tibial and fibular fractures with comminution (Figures 3 and 4). Prior to surgery, all dogs underwent digital radiography, with orthogonal views obtained of the fractured and contralateral limb segment. The time elapsed from when each dog sustained the fracture to initial surgical stabilization ranged from 2 to 3 days. Dogs were given intermittent methadone (0.1–0.2 mg/kg) boluses every 4–6 hours for pain control prior to surgery.

All three dogs were anesthetized using the same anesthetic protocol. Premedication consisted of intravenous dexmedetomidine (3–5 *μ*g/kg) and methadone (0.1 mg/kg); induction was performed with propofol (4–6 mg/kg). Dogs were maintained with inhalant (isoflurane 1.5–2%). Postoperatively, dogs were given intermittent intravenous methadone (0.1–0.2 mg/kg) boluses every 4–6 hours for 24 hours following surgery. Colorado pain scores were assessed every 4 hours following surgery to discharge and used to direct the analgesic protocol.

The MIRIS was used, as previously described by Gilbert et al. [23], to reduce and align the fractures prior to MIPO. Partially threaded 2.8 mm diameter half-pins were inserted in the lateral metaphyseal region of the proximal and distal radius or tibia, allowing for cranial plate application on the radius and medial and cranial plate application on the tibia. Difficulty in seating the half-pin in the proximal radius necessitated placement of the pin in the proximal olecranon in dog #1. The cannulated reduction handles were then slid over the protruding portion of the half-pins until the blunt end of each handle was in direct contact with the cortex of the secure bone segment. The set screw was tightened and the connecting clamps and connecting rod were used to articulate the reduction handles.

Distraction and alignment of the major fracture segments were achieved through manipulation of the MIRIS reduction handles, as traction was applied to the paw. Fracture reduction was initially assessed through palpation, and when reduction was deemed acceptable, the connecting clamps securing the carbon fiber rod and reduction handles were tightened. Sagittal and frontal plane alignment and reduction were then assed intraoperatively using fluoroscopy (Hologic®, Marlborough, MA) and, if necessary, adjustments were subsequently made by loosening the connecting clamps and repositioning the handles on the connecting rod to ensure appropriate fracture reduction and alignment.

**Figure 5:**
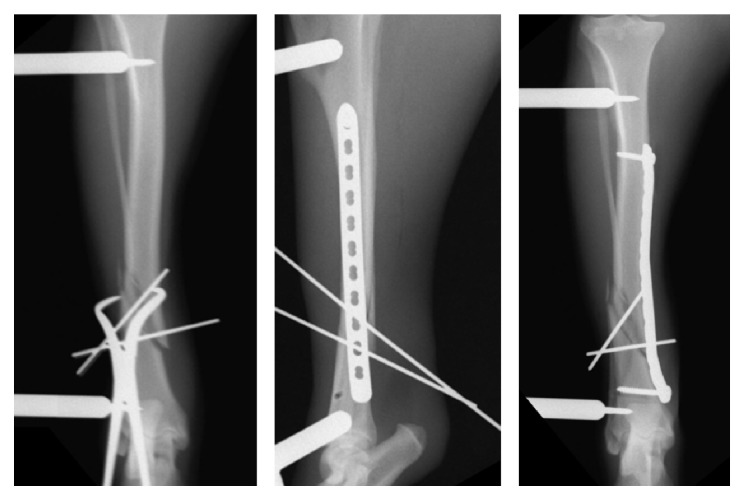
Intraoperative fluoroscopic images demonstrating the use of percutaneous point-to-point reduction forceps and interfragmentary Kirschner wires to maintain temporary fracture stabilization prior to plate placement.

In dog #1, the radius and ulna were initially underreduced and the distal segment was displaced caudally and proximally. The clamps were loosened and the fracture was toggled into position as greater force was applied to separate the reduction handle with traction applied simultaneously to the manus. The clamps were tightened and the ends of both the radial and the ulnar fracture segments were reduced, although mild procurvatum was present. Dog #2's tibia was initially reduced by applying traction to the reduction handles and pes. Reduction was improved by applying reduction forceps with points (DePuy Synthes, Paoli, PA) through two 5 mm incisions to compress the fracture (Figure 5). When reduction was considered acceptable, two percutaneous cranial-to-caudal interfragmentary Kirschner wires were placed, allowing the point-to-point reduction forceps to be removed (Figure 5). Dog #3's tibial fracture was reduced in a similar fashion to dog #2's, but a single 1.0 mm cranial-to-caudal interfragmentary Kirschner wire was used to maintain the reduction as the plate was placed.

Once reduction was considered acceptable, a 2.7 mm locking compression plate (LCP) (DePuy Synthes Vet, Chester, PA) was placed using a MIPO technique to stabilize each of the fractures [24]. The plates were precontoured using radiographs of the intact contralateral limb. Proximal and distal plate insertional incisions were made, cranially over the radius and medially over the tibia, based on the length of the contoured plate, and an epiperiosteal tunnel was created using Metzenbaum scissors. The plates were inserted via the proximal insertion incision and advanced until the end of the plate was positioned in the distal insertion incision. A cortical screw was placed in one proximal and one distal hole in the plate which improved alignment by drawing the engaged bone segments toward the plate. Locking screws were placed in the remaining screw holes except in instances in which a screw needed to be angulated relative to the plate to avoid placing the screw in the fracture (dogs #1 and #3) or the talocrural joint (dog #2). Supplemental String of Pearls (SOP) interlocking plates (Orthomed, Vero Beach, FL) were applied cranially in the two tibial fractures (Table 1) via the original insertional incisions.

All three fractures were well reduced and aligned on the immediate postoperative radiographs (Table 2). Restoration of radial and tibial frontal and sagittal plane alignment was assessed based on measurements obtained from the contralateral intact bone [25]. There was slight varus angulation of both dog #1 and #2's stabilized limb segment. Dog #1's radius was stabilized in slight recurvatum and dog #2's tibia stabilized in slight procurvatum. Radial or tibial length was restored to within 2% of the contralateral intact bone [26, 27].

Plate bridging ratio, calculated as the proportion of plate length over tibial or radial length and expressed as a percentage [27], ranged from 66 to 71%. The plate span ratio, defined as plate length divided by the length of the fracture [19, 27], ranged from 6 to 40%. Plate working length, calculated by measuring the distance between the proximal and distal screws closest to the fracture divided by the length of the stabilized bone segment and expressed as a percentage, ranged from 13 to 41% [27]. The fracture span, the % of the bone length affected by the fracture [19, 26, 27], ranged from 4 to 29%.

All three dogs were placing substantial weight on the stabilized limb when discharged from the hospital 2 days following surgery. Owners were instructed to enforce strict confinement for 1 month, allowing short walks on a leash for purposes of urination and defecation. Dogs were prescribed 7 days of tramadol (3-4 mg/kg q8–12 h), 7 days of cephalexin (30 mg/kg q12h), and 5 days of carprofen (2.2 mg/kg q12h).

Dog #3 returned to the hospital a week following surgery when the owner noted an acute increase in lameness and angulation of the dog's right crus. On examination, the dog had a pronounced right hind limb weight-bearing lameness. The right crus was swollen with obvious valgus angulation. Radiographs showed that the LCP had bent and the SOP plate had broken over the fracture site, resulting in a loss of reduction, valgus angulation, and further fracture comminution (fracture span increased to 37%) (Figure 4). Revision surgery was performed on the following day and the implants were removed through the original plate insertion incisions. The MIRIS was reapplied laterally and the proximal plate insertion incision was extended proximally. After satisfactory reduction and alignment had been obtained using the MIRIS, a contoured 3.5 mm LCP was applied in MIPO fashion (Figure 4). Cortical screws were placed in the third hole from the proximal end of the plate and the hole at the distal end of the plate, drawing the tibia to the contoured plate, which improved frontal plane alignment. To improve craniocaudal fracture alignment, pointed reduction forceps with serrated jaws (DePuy Synthes, Paoli, PA) were placed through the proximal plate insertional incision with one jaw positioned along the cranial cortex of the proximal tibial segment and the tip of the other jaw placed on the caudal border of the plate [22]. The screw in the proximal portion of the plate was loosened and closing the forceps improved alignment by pivoting the distal end of the proximal segment caudally. Four locking screws were placed in the proximal portion of the plate and one locking and one additional cortical screw were placed in the distal portion of the plate. Postoperative radiographs showed that tibial length had been restored to within 1% of the contralateral intact tibia; however, there were 3° of residual valgus and 5° of residual recurvatum angulation.

All three fractures subsequently went on to reach radiographic union without loss of reduction or fixation by 10 weeks following surgery; however, dog #3 developed a draining tract 5 months following surgery, which necessitated plate removal.* Serratia marcescens* was cultured from the plate and screws, and enrofloxacin (5 mg/kg q12h for 14 days) was administered based on the sensitivity results. The draining tract resolved within 5 days of surgery. The owners of all three dogs were asked to return their dogs for reevaluation between 3 and 8 months following surgery and all three dogs had excellent limb function at the time of the final evaluation (median: 237 days; range: 92–238 days). Force plate analysis performed at the final recheck evaluation identified that all three dogs had slight reductions in peak vertical force (PVF) and peak vertical impulse (PVI) in the fractured limb when compared to the contralateral limb (Table 3); however, these reductions were not found to be significant (PVF: *p* = 0.102; PVI: *p* = 0.118) when compared using Student's *t*-test.

Goniometry [28] and circumferential [29] measurements of muscle mass were performed at the final evaluation for each dog (Table 3). Flexion was slightly decreased (median: 5°; range: 3 to 7°) in the joints adjacent to the fracture in all three dogs, with the exception of a moderate decrease of flexion with a difference of 23° identified in dog #3's hock. A mild decrease in extension was measured in the stifle and hock (3° and 4°, resp.) of dog #3. None of these alterations in the range of motion limited limb function. Mild muscle atrophy (range: 2–4 mm) was identified in the brachial and thigh musculature of the fractured limb in dogs #1 and #3, respectively; otherwise contralateral limb muscle mass was symmetrical at other locations measured.

## 3. Discussion

We found that the use of the MIRIS efficiently facilitated MIPO applications in these three fractures. The instrumentation was easy to use and apply and afforded good reduction, with minimal impedance to plate placement. Similar to findings in a cadaveric study [23], seating of the half-pin in the markedly convex, proximal radius can be difficult and proved problematic in dog #1. The half-pin was subsequently placed in the olecranon which allowed effective indirect reduction of the fracture. A smaller version of the MIRIS is available which utilizes 1.6 mm diameter half-pins (DePuy Synthes, Paoli, PA). The smaller system may reduce the problems associated with seating the half-pin in the proximal radius and may expand the use of the MIRIS to include diaphyseal long bone fractures in small dogs, as well as certain metaphyseal fractures which afford a limited area of bone for seating the half-pin.

We inserted the half-pins laterally so the MIRIS would not interfere with plate placement. Lateral application required placing the proximal half-pins through substantial muscle mass, especially in the two dogs with tibial fractures. Liberal release incisions were made to simplify pin placement. The laterally positioned MIRIS primarily facilitated reduction in the frontal plane. With lateral application of the MIRIS, unilateral traction, which occurs when manipulating the reduction handles, has the propensity to induce varus angulation within the limb. In contrast, prior studies evaluating the use of a two-ring circular fixator construct during MIPO application found a tendency to create a slight valgus angulation [7, 23]. The induced varus was 3° or less and did not have appreciable clinical ramifications, but ideally frontal plane angulation should be avoided. To prevent creating varus angulation, particular attention should be paid to ensure the reduction handles do not diverge as traction is applied to the limb. Applying simultaneous traction to the paw was helpful in restoring normal alignment.

All three dogs had spiral fractures which we were able to effectively reduce in an indirect, closed fashion. While the MIRIS is effective in restoring alignment of the fractured limb segment, this device is less adept at providing precise anatomic reduction. Percutaneously applied reduction forceps were used to improve reduction of the two tibial fractures and interfragmentary Kirschner wires were subsequently placed to maintain reduction during plate application [22]. The Kirshner wires were placed in a cranial to caudal orientation so as not to interfere with the medial and craniomedial plate placement. Accurate contouring of the plate and initial placement of a cortical screw in each of the major fracture segments further improved reduction [18–20, 22].

The implant failure that occurred in dog #3 was ascribed to using an undersized, 2.7 mm plate. We had concerns at surgery that the plate was undersized and a second orthogonal SOP plate was applied to increase construct stability [30, 31], but ultimately this fixation was not sufficient. At the revision surgery, a longer 3.5 mm plate, with a consequently larger area moment of inertia and therefore higher bending stiffness [32] than the combined 2.7 mm LCP and 2.0 mm SOP plates, was placed. In addition, during application of the 3.5 mm plate, the screws were placed closer to the fracture, decreasing the plate working length and therefore decreasing plate strain [33, 34]. Use of a 3.5 mm plate at the time of the initial surgery would have likely allowed the fracture to reach union without implant failure and reduced the risk of a surgical site infection. The incidence of surgical site infection following MIPO applications is historically low and has been ascribed to the mitigation of iatrogenic soft tissue trauma and shorter duration of surgery [11–13, 27].

The MIRIS system again provided efficient fracture reduction during dog #3's revision surgery, despite the fracture having been sustained 9 days prior to revision. The system did not impede placement of a longer and larger plate. The fracture was reasonably well aligned following application of the MIRIS. Accurate plate contouring, based on radiographs of the intact contralateral tibia, facilitated frontal plane alignment. Reduction forceps, placed in the proximal plate insertion incision, were used to leverage the proximal fracture segment against the plate and further improve alignment in the sagittal plane. The mild residual valgus and recurvatum present following revision were ascribed to our inability to completely overcome craniolateral soft tissue constraints inherent to reducing a fracture that was sustained 9 days previously.

These cases document our initial clinical experience using the MIRIS for MIPO application in dogs. The MIRIS was easy to apply and consistently resulted in reductions that were near anatomic, with acceptable restoration of length and alignment. Plate and screw placement was unimpeded by the MIRIS, facilitating implant application. Inappropriate implant selection was considered the primary reason for the implant failure experienced by dog #3 and was unrelated to the use of the MIRIS system, as demonstrated by its use during revision surgery with adequate fracture reduction and lack of impedance to a larger plate. Despite initial implant failure and eventual surgical site infection in dog #3, all three dogs had excellent clinical outcomes at the time of final evaluation. Further investigation is required to assess the use of this system for different fracture configurations and locations. Use of the MIRIS system with smaller diameter half-pins and reduction handles for the treatment of fractures in small dogs warrants investigation.

## Figures and Tables

**Table 1:** Clinical information for three dogs with fractures stabilized via minimally invasive plate osteosynthesis facilitated by the use of the Minimally Invasive Reduction Instrumentation System.

Dog	Age (months)	Weight (kg)	Breed	Fracture limb segment	Fracture configuration	Fixation method
1	56	11.5	Mixed-breed dog	Left radius and ulna	Spiral, middiaphyseal (radius) Oblique, middiaphyseal (ulna)	9 holes, 2.7 mm LCP^h^

2	60	17.8	Australian sheepdog	Right tibia and fibula	Comminuted, spiral mid- to distal diaphyseal (tibia) Oblique, distal diaphyseal (fibula)	12 holes, 2.7 mm LCP 10 holes, 2.7 mm SOP^i^ plate

3	48	16.2	Mixed-breed dog	Right tibia and fibula	Comminuted, spiral mid- to distal diaphyseal (tibia) Transverse, middiaphyseal (fibula)	*Initial surgery* 12 holes, 2.7 mm LCP 12 holes, 2.0 mm SOP plate *Revision surgery* 11 holes, 3.5 mm LCP

^h^LCP: locking compression plate; ^i^SOP = String of Pearls interlocking plate.

**Table 2:** Radiographic parameters assessed from the immediate postoperative radiographs in three dogs in which fractures were stabilized via minimally invasive plate osteosynthesis facilitated by the use of the Minimally Invasive Reduction Instrumentation System.

Dog	Fracture span (%)	Radial or tibial length^j^ (mm)	Frontal plane angulation (°)	Sagittal plane angulation (°)	Plate bridging ratio (%)	Plate span ratio (%)	Plate working length (%)	Time to union (weeks)
1	4	120/120	2 (varus)	2 (recurvatum)	70	6	13	10

2	18	170/173	3 (varus)	2 (procurvatum)	66	32	20	9

3 Initial Surgery	29	150/152	None	None	71	40	41	NA
Revision Surgery	37	150/152	3 (valgus)	5 (recurvatum)	97	38	32	8

^j^Fractured bone length/intact bone length; NA: not applicable.

**Table 3:** Clinical parameters assessed at the time of final evaluation in the three dogs in which fractures were stabilized via minimally invasive plate osteosynthesis facilitated by the use of the Minimally Invasive Reduction Instrumentation System.

		Force plate (N)		Limb circumference (mm)				Goniometry (degrees)											
		PVF	PVI	Brachium	Antebrachium	Thigh	Crus	Shoulder		Elbow		Carpus		Hip		Stifle		Hock	
								F	E	F	E	F	E	F	E	F	E	F	E
Dog 1	Fractured	123.64	11.16	151	92	-	-	54	132	23	145	42	184	-	-	-	-	-	-
	Contralateral	131.77	11.92	155	89	-	-	55	130	20	140	35	180	-	-	-	-	-	-

Dog 2	Fractured	74.66	7.94	-	-	330	138	-	-	-	-	-	-	38	168	42	159	38	162
	Contralateral	89.59	13.75	-	-	329	137	-	-	-	-	-	-	32	172	41	152	38	160

Dog 3	Fractured	66.63	6.78	-	-	260	134	-	-	-	-	-	-	24	175	30	165	48	165
	Contralateral	67.02	8.17	-	-	262	136	-	-	-	-	-	-	29	175	23	168	25	169
